# Structural analysis of receptors and actin polarity in platelet protrusions

**DOI:** 10.1073/pnas.2105004118

**Published:** 2021-09-09

**Authors:** Simona Sorrentino, Jose Javier Conesa, Ana Cuervo, Roberto Melero, Bruno Martins, Estrella Fernandez-Gimenez, Federico P. de Isidro-Gomez, Jimenez de la Morena, Jan-Dirk Studt, Carlos Oscar S. Sorzano, Matthias Eibauer, Jose Maria Carazo, Ohad Medalia

**Affiliations:** ^a^Biochemistry Department, University of Zurich, 8006 Zurich, Switzerland;; ^b^Instruct Image Processing Center, National Center of Biotechnology, 28049 Madrid, Spain;; ^c^Division of Hematology, University Hospital Zurich, 8091 Zurich, Switzerland

**Keywords:** actin, cryoelectron tomography, platelets, receptors

## Abstract

During activation the platelet cytoskeleton is reorganized, inducing adhesion to the extracellular matrix and cell spreading. These processes are critical for wound healing and clot formation. Initially, this task relies on the formation of strong cellular–extracellular matrix interactions, exposed in subendothelial lesions. Despite the medical relevance of these processes, there is a lack of high-resolution structural information on the platelet cytoskeleton controlling cell spreading and adhesion. Here, we present in situ structural analysis of membrane receptors and the underlying cytoskeleton in platelet protrusions by applying cryoelectron tomography to intact platelets. We utilized three-dimensional averaging procedures to study receptors at the plasma membrane. Analysis of substrate interaction-free receptors yielded one main structural class resolved to 26 Å, resembling the α_IIb_β_3_ integrin folded conformation. Furthermore, structural analysis of the actin network in pseudopodia indicates a nonuniform polarity of filaments. This organization would allow generation of the contractile forces required for integrin-mediated cell adhesion.

## Significance

Platelet activation induces reorganization of the cell and the formation of cellular protrusions, pseudopodia. These processes are accompanied by remodeling of the actin cytoskeleton and the activation of platelet integrins, which mediate strong adhesion to the extracellular matrix. In this work, we analyzed the actin polarity and integrin architecture in pseudopodia. A nonuniform polarity of actin filaments in pseudopodia indicates that these protrusions may be involved in contractile acto-myosin forces. Heterogeneity in integrin conformation was found, while solely a bent integrin structure was resolved, ∼50 to 70 nm above the support.

Platelets are activated to form a primary adhesion at injured vessels, which leads to their aggregation and clot formation (1, 2). These processes are mediated by the activation of several adhesion receptors and downstream signaling pathways, leading to actomyosin contractility (3–5). These signals include changes in the organization of the cytoskeleton, causing distinct morphological remodeling (6). This process is followed by extensive actin polymerization, forming multiple pseudopodia, filopodia-like structures/microspikes (7).

Through polymerization, F-actin pushes the cell membrane outwards, forming protruding structures, such as lamellipodia and filopodia (8). The organization of the filaments has a major impact on the interaction of the cytoskeletal network with the extracellular matrix (ECM). The mechanism of actin filament remodeling and the polarity of each filament within a platelet is still not clear. Understanding these aspects of the actin network would allow fine-grained modeling of cytoskeletal-based mechanical processes (9). Moreover, integrin-mediated cell adhesion of these cellular protrusions to the ECM, in addition to contractile force generation by myosin II (10), are crucial for platelets adhesion and the wound-healing process (11).

A major family of platelet adhesion receptors (i.e., integrins) mediate a firm adhesion to exposed ECM molecules of endothelial lesions, leading to platelet contraction via binding of soluble integrin ligands, such as fibrinogen and plasma fibronectin (12). Since platelet integrins are continuously exposed to their soluble ligands, their activity must be tightly controlled to avoid deregulated thrombus formation. The integrin α_IIb_β_3_ is the most abundant platelet integrin receptor, initiating platelet attachment to fibrinogen (13).

Structural analysis of integrin receptors has been conducted mainly using purified receptors (14–16). Atomic resolution structural determination of the extracellular domains by X-ray crystallography provided fundamental information on integrin organization, while NMR spectroscopy of the intracellular tail and transmembrane domains resolved the organization of the membrane domain (17, 18). Two-dimensional (2D) and three-dimensional (3D) negative-stain electron microscopy provided information on the active and inactive conformations of the receptor (14, 19). Integrin activation models based on these studies suggest that the integrins have at least two conformational states: a low-affinity state, where the protein is in a bent position, imaged with X-ray crystallography (15), and a high-affinity state, where the receptor is thought to be in an extended position. More recently, the structure of the α_IIb_β_3_ headpiece in complex with an antagonist was resolved by cryoelectron microscopy (cryo-EM) (20), further hinting at the integrin mode of function. However, despite this progress, contradicting views on the mechanism of integrin activation remain.

The receptor structure has been further studied in its hydrated state by single-particle analysis, where the full-length protein was reconstituted in nanodiscs (21). That study demonstrated it is possible to observe extended and bent conformations, along with two intermediate conformations. The authors suggested that the integrin is in equilibrium between these conformations, and they observed that the presence of talin head domain and RGD peptide changes the relative proportion of the receptor in these conformations. This evidence proposes that the presence of physiological binders and the receptor’s environment may influence its conformations, thus indicating the necessity of resolving the integrin structure in situ.

Here, we studied the structure of receptors, in situ, using human platelets spread on EM grids in the presence of divalent ions. For that purpose, we utilized cryoelectron tomography (cryo-ET), and its ability to acquire in situ structural information using intact cells. Since platelets are thin specimens lacking a nucleus, they are suitable for cryo-ET. Additionally, the α_IIb_β_3_ integrin heterodimer is highly abundant, with 100,000 receptors per platelet (22). These factors make platelets ideal targets for this study. Structural information was obtained through subtomogram averaging and classification, providing insight into the structure of the most abundant platelet receptor. Additionally, we analyzed the cytoskeletal architecture of intact mouse platelets using cryo-ET. The tensile actomyosin cytoskeleton exerts forces, through integrins, onto the ECM, and thereby plays a major role in platelet spreading and contraction (23). Our analysis of the cytoskeleton architecture and actin polarity revealed a nonuniform polarity of actin in pseudopodia platelet protrusions.

## Materials and Methods

### Purification of Human Platelets.

This study was approved by the University of Zurich Hospital ethics board. Blood was obtained from healthy donors (*n* = 5); all donors provided informed consent. Blood was collected in tubes containing 0.375 mL of Citrate-Mix B (179 mM Citric acid monohydrate, 154 mM Trisodium citrate dihydrate, 267 mM Glucose, pH 5.1, BD Vacutainer blood collection tubes). Blood was centrifuged at 180 × *g* for 15 min at room temperature and the yellow upper phase, platelet-rich plasma (PRP), was collected. The purified PRP was supplemented with citric acid (5 mM) and centrifuged at 900 × *g* for 5 min. The platelet-containing pellet was resuspended in Tyrode’s buffer (134 mM NaCl, 12 mM NaHCO_3_, 2.9 mm KCl, 0.34 mM Na_2_HPO_4_, 10 mM Hepes, 5.5 mM glucose). Freshly purified platelets were immediately used for EM grid preparations.

### Preparation of EM Grids.

Gold grids coated with a Silicon membrane (SiO_2_) mesh (R 1/4, 200 mesh, Quantifoil) were functionalized with 50 μg/mL Fibrinogen (from human plasma, Merck 341576) for 1 h at 37 °C, and then washed twice with Tyrode’s buffer. Platelets were seeded on the functionalized grids either in presence of 1 mM Ca^2+^ and 1 mM Mg^2+^ or in presence of 1 mM Mn^2+^. Ca^2+^ and Mg^2+^ were used to promote platelet spreading, A drop of 4 μL BSA-coated 10-nm fiducial gold markers (Aurion) was applied on the EM grids prior to plunging them into liquid nitrogen cooled liquid ethane.

### Data Acquisition and Tomographic Reconstruction.

Data acquisition was performed using a FEI Titan Krios transmission electron microscope equipped with a quantum energy filter and a K2-Summit direct electron detector (Gatan). The microscope was operated at 300 keV in zero-loss mode with an energy filter slit-width set to 20 eV. A total of 30 tomograms of the spreading platelets (incubated with Ca^2+^ and Mg^2+^ ions) were acquired at 8-µm underfocus, in a 0.34-nm pixel size at the specimen level. This first dataset was collected using the SerialEM software (24), covering an angular range from −60° to +60° with 2° increments. The cumulative electron dose was ∼60 e^−^/Å^2^. Additionally, 25 tomograms of the platelets were acquired at 4-µm underfocus, in a 0.22-nm pixel size. In this case, an angular range of −60° to +60° was covered with 3° increments, and the cumulative electron dose was ∼80 e^−^/Å^2^. All image stacks were down-sampled and motion corrected using the MotionCor_driftcorr software (25). Next, CTF correction of the tilt-series was applied as previously described (26), prior to back-projection reconstruction as implemented in MATLAB using the TomToolbox package (27).

### Actin Polarity Determination.

Actin polarity determination was conducted using the Actin Polarity Toolbox (APT), as described in refs. 28 and 29. Actin filaments were initially segmented from six tomograms using Amira (ZIB and FEI SAS Thermo Fisher Scientific). Using these segmentations, 3D coordinates of actin segments were selected along the filaments with a spacing of 8 nm. Using these coordinate lists, actin segments were reconstructed into subtomograms composed of 168 × 168 × 168 voxels, corresponding to a box size of 37 nm. The 3D coordinate extraction from segmentations was performed using a script written in MATLAB (Mathworks) and the subtomogram reconstructions were conducted using tom_toolbox (27).

Next, central volumes (11 nm in thickness) of the subtomograms were masked and projected onto a common plane, along the *z*-direction. The 2D projections were further aligned to produce 2D classes that were used for 3D reconstruction by the helical toolbox of RELION (30). We used a helical rise of 27.6 Å and the helical twist to 166.7° (31). The 3D reconstruction was conducted using in-plane angle and translation determined during the 2D classification step. Of an initial total of 60,289 particles from 120 classes, 25,327 particles from 22 classes were selected for 3D reconstruction. We verified the resolution measurement, 14 Å, by determining the FSC (32) using the gold-standard criteria as implemented in RELION.

Mapping back the actin segments into the 3D cellular volumes to form the corresponding individual filaments and the subsequent visualization of those filaments was conducted in a MATLAB program, part of the APT (28). All underlying data summarized in the plots (confidences scores) were obtained using MATLAB program, also part of the APT.

All segmentations, actin structures and filaments were visualized with University of California San Francisco Chimera (33) or Matlab (TomToolbox).

To determine the actin polarity in all 28 pseudopodia tomograms, they were first segmented automatically using a previously published method based on convolutional neural networks (34). All subsequent steps remained similar to the procedures described above. The stacked column charts (see Fig. 3*B*) were created using Microsoft Excel.

### Platelet Receptor Structural Determination.

We used an automatic coordinate selection procedure on the second acquisition data set (4-µm underfocus and 0.22-nm pixel size). CTF was estimated using CTFPLOTTER software from imod (35), and the tomograms were reconstructed using NovaCTF software (36). Membranes were segmented in tomograms using tomosegmemtv (37). Next, segmented tomograms were submitted to PySeg software (38) in order to detect densities attached to the membrane, at position that the membrane is well resolved (∼50 to 70 nm above the support). This software consists of an automatic particle picking procedure to select densities connected to the plasma membrane. A total of 73,950 subvolumes with their corresponding orientations were reconstructed and averaged to render an initial model. Subtomogram averaging was performed using RELION (39) inside Scipion3 (40). The subtomogram averaging procedure was developed in different stages (*SI Appendix*, Fig. S4). In a first step, exhaustive 3D classification was performed yielding a 3D class that could correspond to a known bend integrin conformation (PDB ID code 3FCS). In a second step, a more restrictive subvolume selection using PySeg (tilt angle filtering) (38) was conducted and the subtomogram averaging procedure was repeated. In a final alignment and classification step, we used the 40-Å filtered average resulting from the first stage as an initial model. The final volume was reconstructed with 5,185 subtomograms resulting in a resolution around 26 Å at 0.5 criteria (*SI Appendix*, Fig. S5).

Initial docking of the bent integrin receptor structure (PDB ID code 3FCS) was performed using Chimera software (33). Then, the density corresponding to the integrin was subtracted and subjected to flexible fitting using imodFit (41). Final volumes were mapped back into selected tomograms using map back subtomograms protocol inside Scipion3.

## Results

The application of cryo-ET allows the reconstruction of 3D density maps of unperturbed cellular environments at a resolution of 2 to 3 nm (42–44). Therefore, single-actin filaments and proteins can be readily detected in tomograms of eukaryotic cells (45–47).

### Imaging Platelets on EM Grids.

**Fig. 1.**
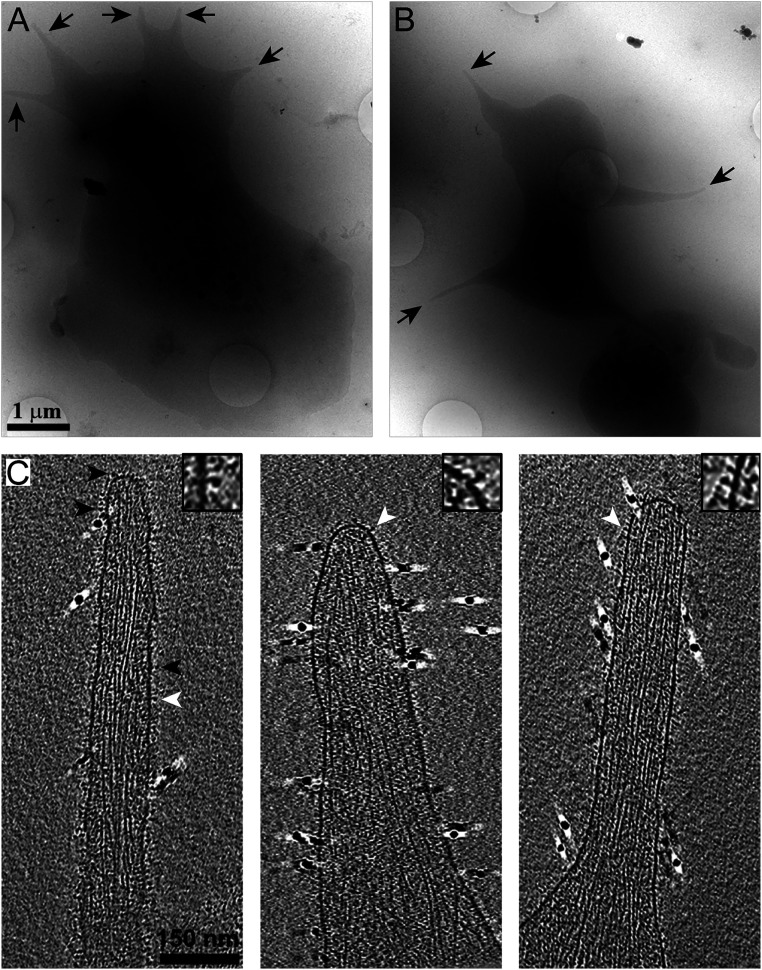
Cryo-ET of platelet pseudopodia. (*A* and *B*) Cryo-EM of human platelets spread on fibrinogen-functionalized SiO_2_-coated gold grids. Pseudopodia are detected in single projections (arrows). (*C*) *x*-*y* slices, 10 nm in thickness, through cryotomograms of pseudopodia. The membrane is decorated with protein densities (arrowheads) while the cytoskeleton is seen in the cytoplasm. Insets show magnified membrane receptors (white arrowheads). Fiducial gold clusters are seen as 10-nm black densities.

Platelets are small cells that readily spread to form thin areas that are suitable for in toto investigation by cryo-ET, therefore not requiring additional sample preparation steps (48–50). Cryo-EM imaging of spread platelets allows simple detection of most cellular regions, covering 10 to 20 µm^2^ (Fig. 1 *A* and *B*). During initial spreading, platelets form a large number of filopodia-like pseudopodia protrusions that can be detected in low-magnification cryo-EM images (Fig. 1 *A* and *B*, arrows). In a previous study, we showed that the concentration of the GpIb receptor, an abundant receptor, is reduced in pseudopodia (51). Consequently, studying these protrusions would provide a further enrichment of integrins, over other platelet receptors. Here, we have focused our analysis on these thin cellular protrusions <100 nm in thickness.

**Fig. 2.**
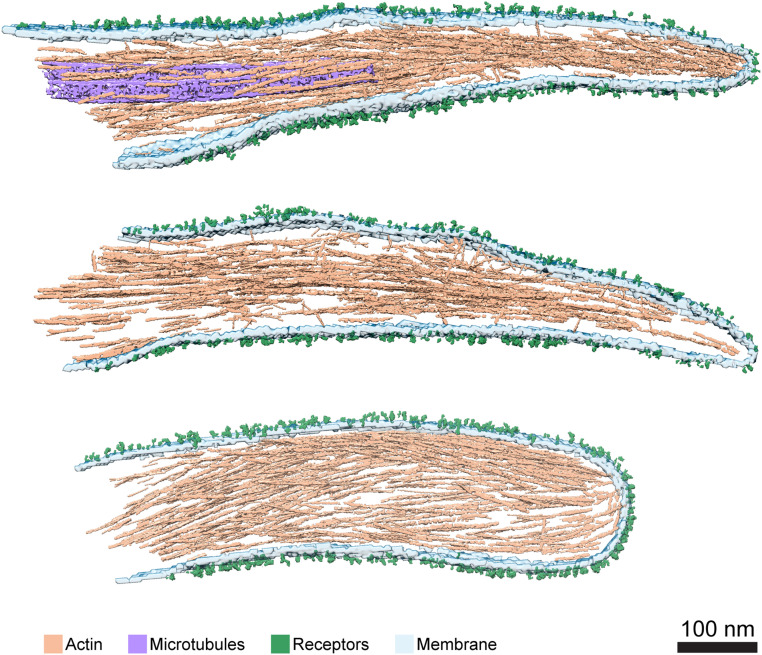
The 3D architecture of platelet pseudopodia. Surface rendering of three pseudopodia. Three of the volumes were rendered and all elements composing pseudopodia are shown. Actin (light brown), microtubules (purple), membrane (light turquoise), and receptors (green) were segmented. (Scale bar, 100 nm.)

Analysis of cryotomograms revealed densities attached to the extracellular phase of the plasma membrane (Fig. 1*C*, arrowheads, *Inset*), while the cytoplasm contains cytoskeleton filaments (i.e., actin filaments and often microtubules), as previously described (52, 53). Cryo-ET analysis of mouse platelets deficient in the α_IIb_β_3_ integrin (54) shows a smooth membrane lacking extracellular densities (*SI Appendix*, Fig. S1), similar to the membrane appearance of platelet-derived microparticles, purified from Glanzmann thrombasthenia patients (55). Moreover, previous gold labeling of platelets in conjunction with cryo-ET indicated the appearance of integrins attached to the plasma membrane (50, 56). These observations confirm that many of the densities that are emanating from the plasma membrane correspond to the α_IIb_β_3_ integrins (Fig. 1*C*). Surface-rendering views of three protrusions are shown in Fig. 2 (the respective tomograms are shown in Movies S1–S3). The spatial organization of the pseudopodia are visualized and the bundled actin filaments (Fig. 2, light brown), microtubules (Fig. 2, purple), the plasma membrane (Fig. 2, light turquoise), as well as the receptors (Fig. 2, green) at the plasma membrane were detected. A high density of actin filaments is typical of these protrusions.

### Actin Organization and Polarity in Platelet Protrusions.

Actin polymerization is a fundamental process that facilitates cell motility, at the leading edges of cells as well at other cellular protrusions (57, 58) and processes. Analysis of filopodia protrusions suggested that actin is organized in a parallel manner with uniform polarity (i.e., the barbed end of all filaments point toward the cell membrane) (59). Previously, filopodia protrusions were studied by means of cryo-ET, although due to limited resolution (60), actin polarity could not be determined. To analyze the actin polarity in pseudopodia protrusions of platelets, we employed the APT, which allows analysis of the structure and polarity of individual actin filaments, inside cells (28).

**Fig. 3.**
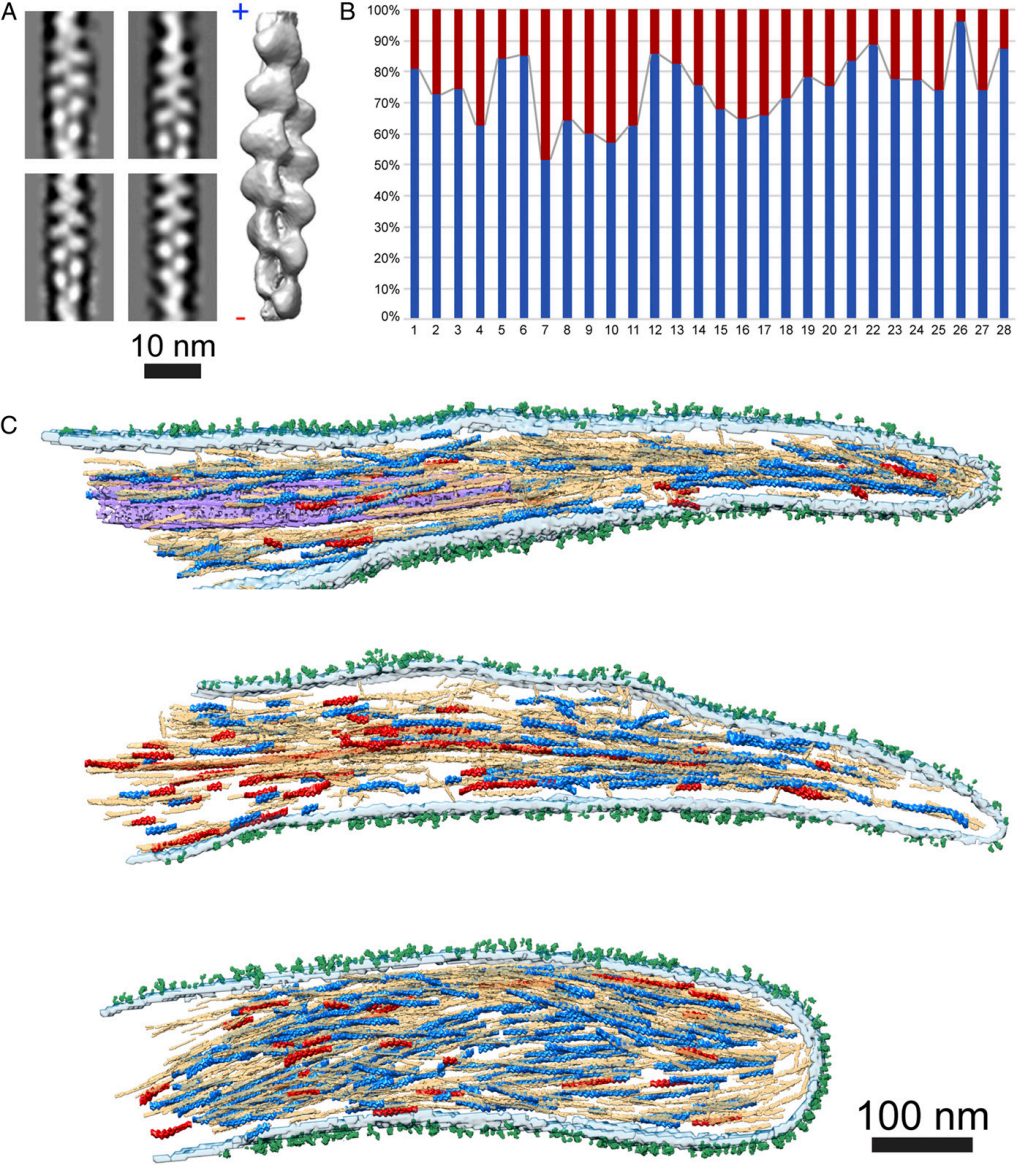
A nonuniform actin polarity in pseudopodia. (*A*) Four 2D class averages of the in situ actin segments (*SI Appendix*, Fig. S2). A 14-Å resolved actin structure (shown in gray) allows identification of actin polarity, barbed (+) and the pointed (−) ends are indicated. (Scale bar, 10 nm.) (*B*) Back-mapping of the actin structure into the tomogram revealed a mixed actin polarity in each tomogram. Actin filaments pointing to the pseudopodia tip (blue) were found to compose 74% ± 10% of the total filament numbers, while actin filaments pointing toward the cell body (red) represent 26% ± 10%. (*C*) The pseudopodia shown in Fig. 2 were used to overlay the polarity of the total actin filament content within protrusions. Actin filaments and segments pointing to the plasma membrane are colored in blue while the actin pointing toward the cell is colored in red. The total segmented actin filaments are depicted in light brown. Additionally, microtubules (purple), membrane (light turquoise) and receptors (green) were segmented as well. (Scale bar, 100 nm.)

Here, we acquired and reconstructed 28 tilt series of pseudopodia, and applied the APT procedure (*Materials and Methods*). The polarity of actin can be readily resolved through the characteristic appearance of the 2D class averages (Fig. 3 *A*, *Left*). The structure of actin, shown in Fig. 3*A* (*SI Appendix*, Fig. S2), was resolved to 14 Å (*SI Appendix*, Fig. S3*A*). Next, we mapped back the filaments into their original position (i.e., into the cellular context), allowing us to determine the polarity of individual filaments within the platelet pseudopodia. We applied a statistical confidence analysis on segments within individual filaments to verify the correctness of our assignment (*SI Appendix*, Fig. S3 *B* and *C*), which indicated that the polarity of ∼74% of the filaments could be determined with sufficient confidence.

In contrast to filopodia, actin filaments in pseudopodia exhibit a nonuniform polarity organization. Within the polarity-resolved filaments in single pseudopodia, we found that 74% ±10% of the actin filaments point their barbed end toward the tip of the protrusion while the other 26% ±10% are oriented with the opposite polarity (Fig. 3*B*) (a more stringent statistical criteria, as described in ref. 28, yielded 80% ±11% and 20% ±11%, respectively). These mixed polarity filaments are not concentrated at a unique, constrained position in pseudopodia but rather spread throughout the whole protrusion volume. Fig. 3*C* shows the same three protrusions as in Fig. 2, in which the polarity of the actin was mapped back to the volumes. These results suggest that platelet protrusions are atypical filopodia-like structures that may be involved in contractile acto-myosin forces, requiring a mixed polarity actin network, as in focal adhesions (28).

### Structural Analysis of Receptors in Platelets.

We acquired a dataset using platelets that were cultured on fibrinogen-coated EM grids, in the presence of Ca^2+^ and Mg^2+^ cations (*Materials and Methods*). Due to technical limitations, termed “the missing wedge” effect, the bottom and top membranes of the cell cannot be seen in cryo-tomograms (61). Therefore, we focused this analysis on receptors that are located at the sides of pseudopodia, where the membrane is oriented parallel to the electron beam, although at these regions the receptors are likely not to be in a direct interaction with the fibrinogen coated surface.

**Fig. 4.**
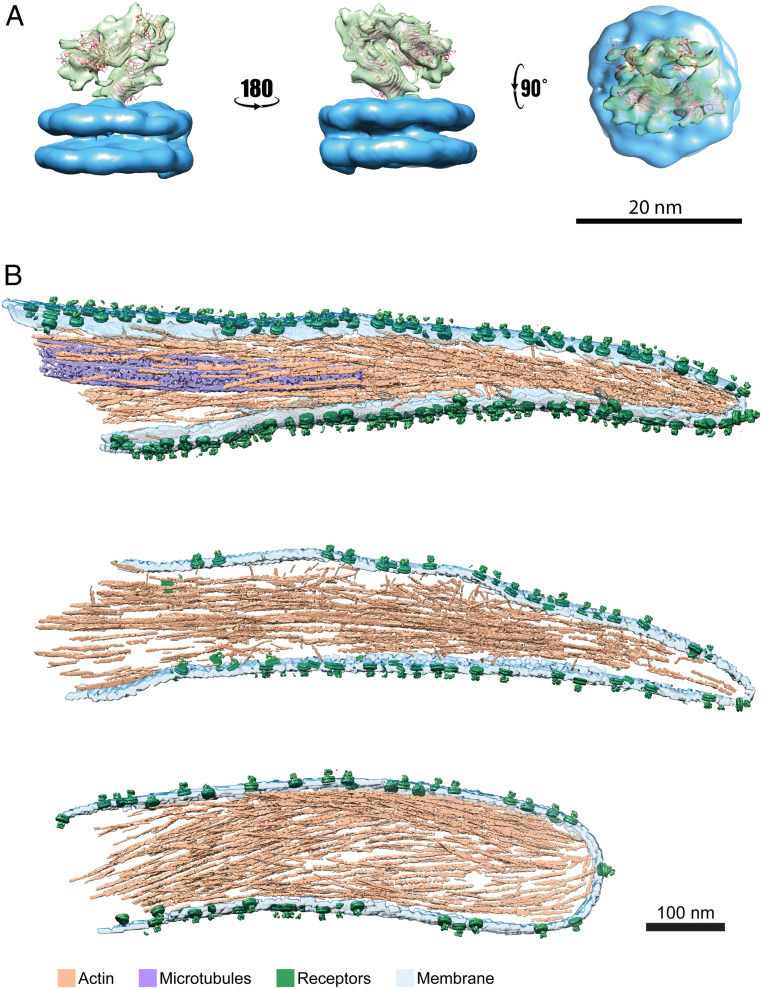
Structural analysis of platelets receptors. (*A*) Subtomogram averaged map of platelet receptors in different orientations, the membrane was colored in blue and the protein receptor in green. The bent integrin structure (PDB ID code 3FCS, in ribbon) was fitted into the density map obtained by in situ structural determination. (*B*) Surface rendering of three pseudopodia where subtomograms corresponding to the platelet receptor (green) were placed back in the original tomograms using the refined coordinates and orientations. Actin (light brown), microtubules (purple) and membrane (light turquoise). (Scale bar, 100 nm.)

Initially, densities attached to the membrane were automatically picked using PySeg software (38). About 74,000 coordinates with their corresponding orientations were selected along the plasma membrane. The reconstructed volumes were averaged, considering their expected normal-to-membrane orientations, to generate an initial model. Subtomograms were than subjected to 3D averaging procedures, using RELION (39) within a Scipion3 framework (40). Classification of the subtomograms (*SI Appendix*, Fig. S4) resulted in a single well-defined class, which contained ∼5,000 subtomograms of ∼74,000 selected, in agreement with the receptors high heterogeneity observed in the cryotomograms (Fig. 1*C*) (55). It reflects at the final averaged structure, which is limited to 26-Å resolution (*SI Appendix*, Fig. S5). The model of the reconstructed receptor shows a 12-nm-tall protein structure (Fig. 4*A*, green) attached to the double bilayer membrane patch (Fig. 4*A*, blue). The receptor presented a folded shape, resembling the proposed low-affinity conformation of integrins (62, 63). Our structure appears more open than the previously determined low-affinity structure; specifically, the head domain is further apart from the “leg” of the integrin as indicated by flexible fitting. Fitting the crystal structure of the extracellular domain of α_IIb_β_3_ (14, 64) in its fully bent conformation indicated similarities with a cross-correlation coefficient value of 0.72 (Fig. 4*A*), suggesting that the reconstructed volume corresponds to an in situ membrane-bound bent integrin receptor.

Finally, we mapped back the original positions of the reconstructed volumes into the pseudopodia-rendering volumes. The position of the integrins showed no apparent preferential distribution or pattern on the pseudopodia membrane of the platelets (Fig. 4*B*). The integrin receptors were homogeneously distributed through the 30 analyzed tomograms (*SI Appendix*, Fig. S5*C*).

## Discussion

Integrin-based platelet adhesions mediate the interactions of the actin cytoskeleton with the ECM through integrin receptors, enabling adhesion to blood vessel injuries and subsequently the formation of a clot. The adhesive properties of platelets are tightly regulated to ensure that the cells are readily activated under appropriate conditions to prevent blood loss in cases of vascular injury. At the same time, undesired adhesion, which can lead to thrombotic events, has to be avoided. The events that lead to integrin activation and adhesion are complex. These events are regulated by the integrin “adhesome,” which is composed of many scaffolding and signaling components (e.g., talin, FAK, kindlin) (65). Specifically, during platelet adhesion, talin changes its conformation to enable binding to the β3 cytoplasmic tail (66), and recruits and activates additional force-sensing proteins, such as vinculin, leading to a series of molecular interactions involving adhesome scaffolding. Alongside these intracellular rearrangements, the current model proposes that α_IIb_β_3_ integrin also undergoes major conformational changes (15, 21), which are needed for signaling and adhesion formation, even though such conformational changes have never been observed under near-native conditions. To better comprehend platelet adhesion, the structure of the integrin receptors as well as the underlying cytoskeleton should be studied, in situ. Therefore, we applied cryo-ET as a tool to study intact platelets.

Analyzing receptors in situ is challenging because of heterogeneity in receptor populations. In platelets, the most abundant receptor is the integrin α_IIb_β_3_. However, platelets contain a multitude of other membrane receptors involved in adhesion, such as the α_2_β_1_, α_6_β_1_, and α_v_β_3_ integrins, the collagen receptors GPV, and P-selectin (67). While classification approaches can eliminate many of these other receptors due to their low molecular weight and dimensions, other integrins may initially be present in our analysis. However, due to their low abundance, these receptors contribute little, if at all, to the resulting structure. Indeed, we analyzed ∼74,000 subtomograms, from which ∼5,000 subtomograms converged into a medium-resolved structural class, resembling the α_IIb_β_3_ bent conformation.

Previously, the structure of this full-length integrin was resolved to a modest resolution (21). These analyses have shown the complexity of the structure and heterogeneity in the receptor conformation, as well as their orientations in respect to the membrane (21, 68). Moreover, a structural study on platelet-derived microparticles supports these findings, showing that chemical or thrombin-mediated activation of the receptors does not have an additional subsequent effect on the receptor’s conformation in divalent ion containing buffers (55). Here, we analyzed receptors that emanate from plasma membranes, perpendicular to the plane of the support and ∼60 nm above it. In these regions both the receptors and the membrane are well visualized by cryo-ET. However, due to the distance above the support, it is unlikely that these receptors interact with the fibrinogen on the support. The receptors are flexible and may exhibit variety of conformations; however, a bent α_IIb_β_3_ integrin conformation was resolved to a resolution of 26 Å. The heterogeneity of the integrin receptors stem from the flexible linker that bridge the transmembrane domain to the ectodomain, at the “tail” of the integrin receptors. Rotation and change in angles at this position was also detected in purified receptors (21), although it may be more pronounced in the bent integrin conformation. Additionally, the resolved folded state of the integrin is more open than the in vitro low-affinity –determined structure, emphasizing the role of the native environment and membrane play in integrin conformation. The change of angles between the tail and the head of the ectodomain (69) also indicates that the α_IIb_β_3_ integrin receptors are felxible and can adopt different conformations, as suggested by Hanein and Volkmann (68). These presumably ECM-free conformational changes may suggest that activation of platelet integrins is spatially and temporally regulated to avoid undesired integrin activation. However, determining the structure of nonpurified integrins, interacting with the ECM, would clarify the flexibility of integrin structures and the accompanied conformational changes.

Platelet shape changes accompany the activation of the acto-myosin contractile system (12, 70). When exposed to divalent ions, shape change begins with a contractile event, that is followed immediately by a burst of pseudopodia protrusions. Here, we analyzed the actin filaments at these protrusion sites (Fig. 1) and revealed their polarity, providing a glimpse of the cytoskeletal organization in pseudopodia protrusions (Figs. 2 and 3).

Although platelets typically have very thin cellular bodies, imaging actin filaments in high resolution is more challenging than in fibroblasts. While the analysis of focal adhesions in mouse embryonic fibroblasts resulted in determining the polarity of 84% of the actin filaments (28), here only 74% of the filaments passed our confidence analysis. The use of SiO_2_-coated EM grids may contribute to the lower contrast of actin filaments; however, platelets tend to spread better on silica membranes than on the conventional carbon-coated EM grids. The difference in contrast may also suggest that the platelet’s cytoplasm is densely packed, leading to increased background and reduced signal-to-noise ratio. Despite the lower contrast, we could determine that within the polarity resolved filaments, the majority of filaments (∼74%) are organized with their faster growing barbed ends oriented toward the tip of the protrusion, a putative adhesion site (Fig. 3*B*). However, ∼26% of the filaments exhibit opposite polarity, suggesting that these protrusions have the capability to form a contractile actin network that may develop into adhesion sites. These findings are in accordance with actin organization in dendritic filopodia that often develop into dendritic spines (71).

Platelets interact with the ECM and apply contractile forces that remodel both the actin cytoskeleton and the ECM. Platelet pseudopodia have been shown to bind fibrin and contracts ECM fibers (72). This process requires a mixed polarity of actin filaments, as we detect in our study, confirming the contractile ability of pseudopodia. Moreover, microtubules are often found in the pseudopodia protrusions, in addition to the actin filaments. This observation is in agreement with a recent study that identified microtubules in over 30% of platelet protrusions (53).

Progress in the application of cryo-ET to receptor protein complexes opens exciting new opportunities for in situ structural biology, in particular allowing the possibility to reconstruct other receptors, such as those of the EGFR family. The development of new software tools (38) and novel sample preparation techniques would allow higher resolved in situ structural determination, approaching atomic resolution. These developments could aid in obtaining a high-definition adhesion map of platelet receptors and the precise conformation of each integrin at adhesion sites.

## Supplementary Material

Supplementary File

Supplementary File

Supplementary File

Supplementary File

Supplementary File

## Data Availability

Structural data that support the findings of this study has been deposited in the Electron Microscopy Data Bank https://www.ebi.ac.uk/emdb/ (accession code EMD-12285). Cryo-ET data has been deposited to the Electron Microcopy Public Image Archive Repository, https://www.ebi.ac.uk/empiar (accession code EMPIAR-10636). All other study data are included in the article and supporting *SI Appendix*.
