# Supplementary material for: Structural analysis of receptors and actin polarity in platelet protrusions
Source: Proc Natl Acad Sci U S A. 2021 Sep 9;118(37):e2105004118. doi: 10.1073/pnas.2105004118 (PMC8449362; doi:10.1073/pnas.2105004118)
Supplement: Supplementary File [file pnas.2105004118.sapp.pdf]

Supplementary Information

Supplementary Figures

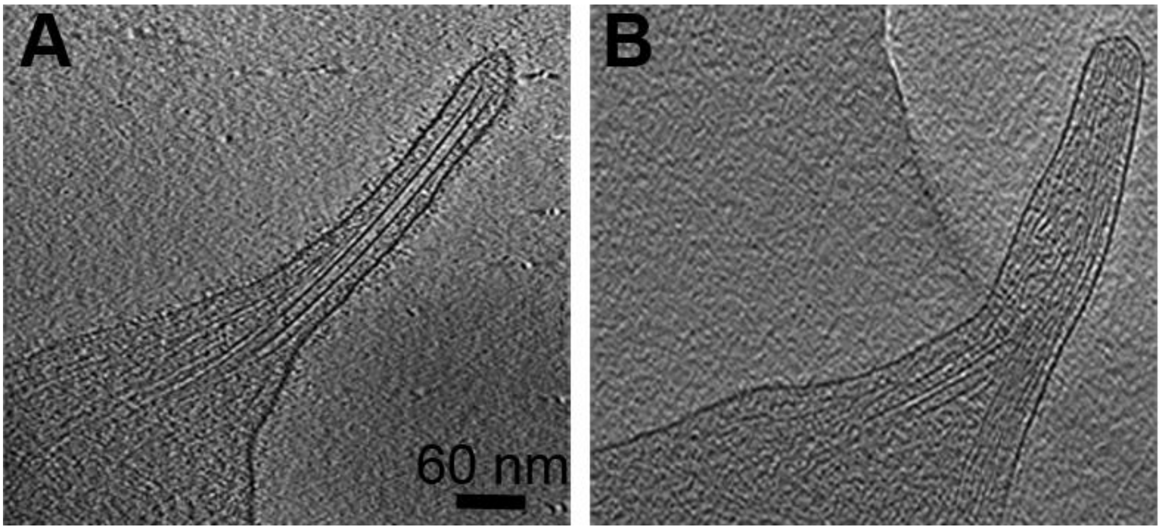

Supp. Figure 1. Electron densities around pseudopodia are $\alpha_{IIb}\beta_3$ integrin expression dependent. A. A 12nm thick x-y slice through a wildtype human platelet. B. An x-y tomographic section through a pseudopodia of mouse platelets expressing no $\alpha_{IIb}\beta_3$

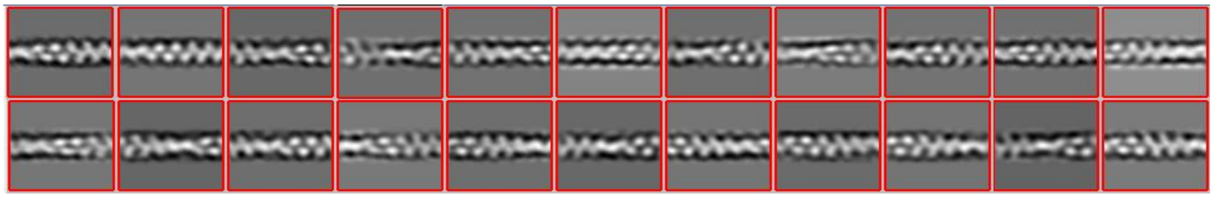

Supp. Figure 2. Structural analysis of actin filaments. The 22 classes used for reconstruction of the actin structure (Figure 3A) and subsequent polarity analysis of individual filaments. Box size 37 nm.

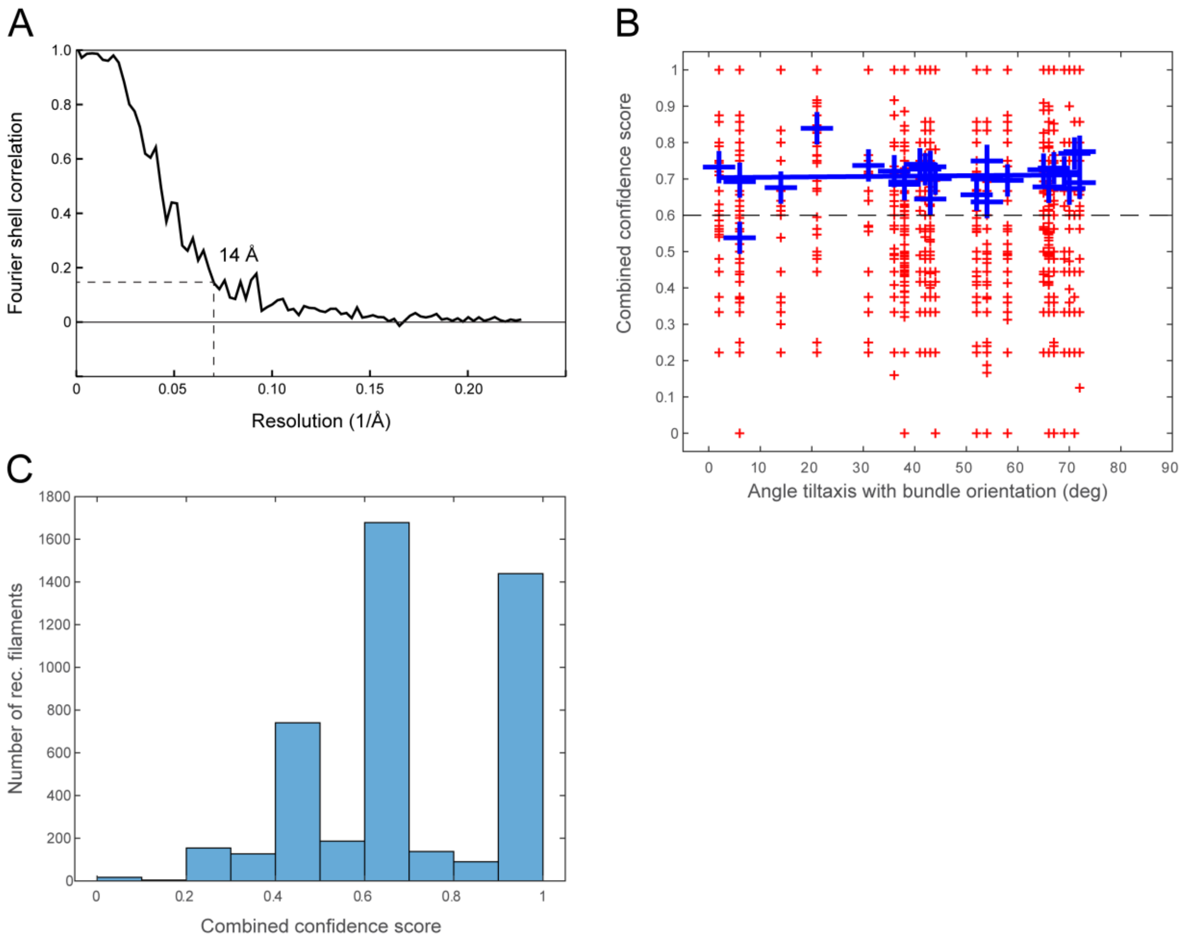

Supp. Figure 3. Structural analysis of actin filaments. A. The *in situ* reconstructed actin filament structure was determined to ~14 Å, using the gold standard criteria, RELION 3.0. **B.** Plot showing the respective ccs values (y-axis). Each red cross marks the ccs of one filament, and the scores are plotted as a function of the bundle orientation in respect to the tilt axis. The blue crosses indicate the average of all ccs values of the filaments implicated in the same actin bundle, and a regression line was fitted to these values (blue line). **C.** Histogram of the respective confidence analysis value of all filaments. Only filaments reaching a ccs ≥ 0.6 (dashed line, to be added) were accepted and shown in Figure 3 (for details see ref. **(1)**) .

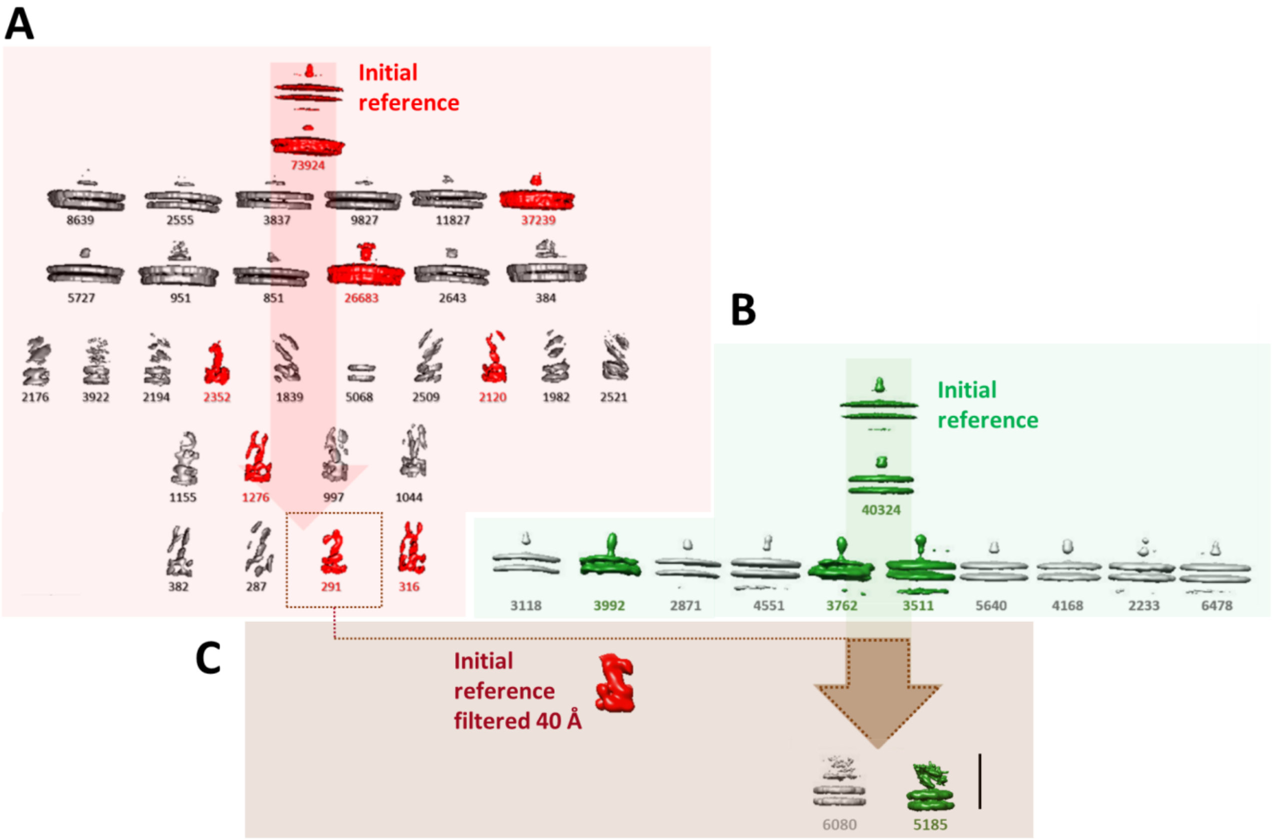

Supplementary figure 4. Subtomogram averaging workflow. Scheme showing the different rounds of 3D classification of the particles selected as receptors bound to the membrane. Positive results were selected when densities are attached to the membrane and they are colored in red and green. Selected classes were used to proceed to the next round of classification. Below each 3D class is the number of subtomograms it contains. The flow diagram is composed of three stages. In stage **A** (red arrow) an exhaustive picking was made using pySeg. The selected subtomograms were averaged in their initial orientations to generate an initial model. 3D classification end with two classes that were selected as positive results. In a second stage, **B**, a new set of subtomograms were selected from the same tomograms used in A using PySeg with more restrictive parameters**.** The initial reference corresponds to the average of the selected subtomograms which were classified to achieve a subset of classes of membrane-bound density maps (green). In a third stage, **C** (brown arrow) a 40 Å filtered class from stage **A**, which resembles a known conformation of the integrin receptor, was used as initial model to classify the subtomogrmas of stage B using RELION, resulting in the final map in green. Scale bar 22nm.

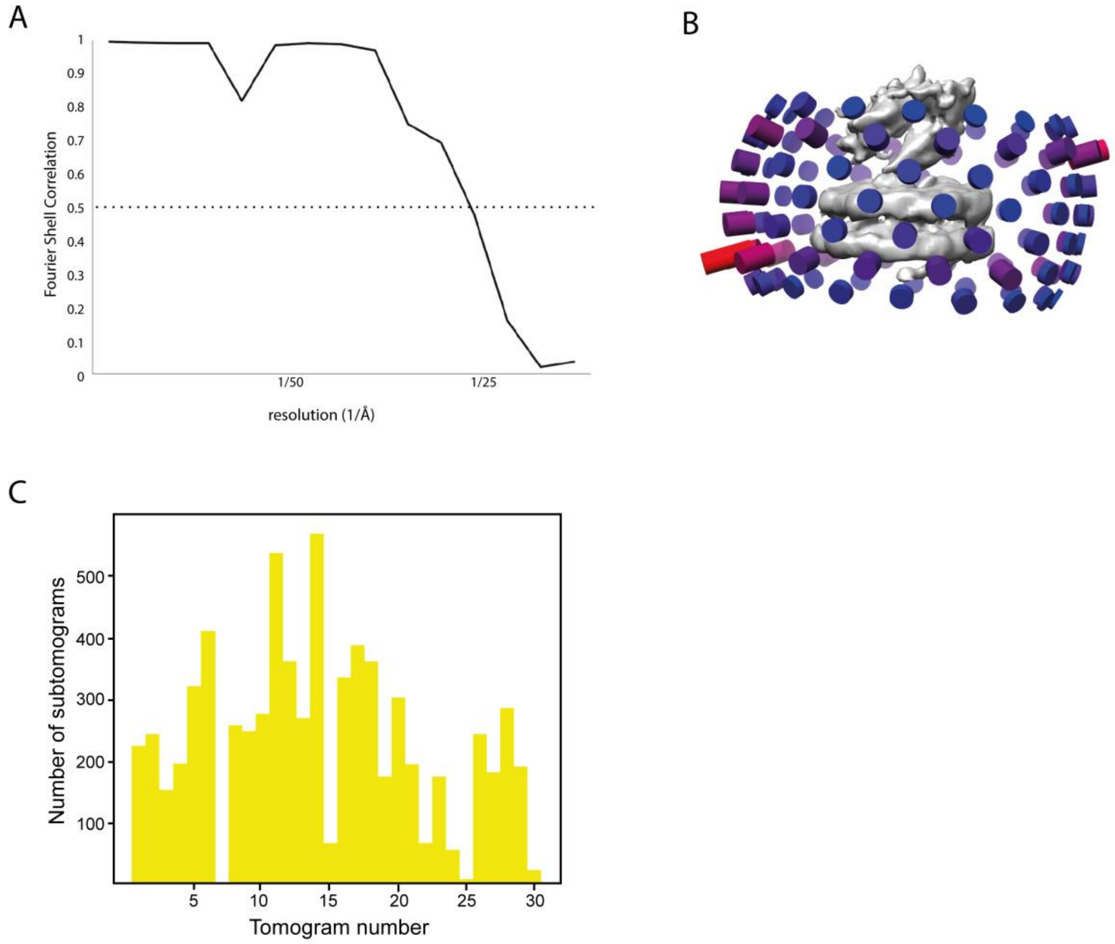

Supplementary figure 5. Structural analysis of platelets receptors. A. The *in situ* reconstructed of folded αIIbβ3 integrin receptor was determined to ~25 Å resolution. **B.** Representation of the different orientations corresponding to each of the subtomograms used to reconstruct the model shown in Figure 4A. Bars in color indicate the representation, number of subtomograms, of the different orientations from blue, low representation, to red, high representation. **C.** Histogram showing the subtomogram distribution across the 30 analyzed tomograms.

Movie_1. Scrolling through the raw-tomographic volume of the pseudopodia shown in Figure 2 (up)

Movie 2. Scrolling through the raw-tomographic volume of the pseudopodia shown in Figure 2 (middle)

Movie 3. Scrolling through the raw-tomographic volume of the pseudopodia shown in Figure 2 (bottom)

Movie 4. The averaged structure shown in Figure 4A is rotated. Membrane in blue, and receptor in green. The bent integrin structure (PDB 3FCS, in ribbon) was fitted into the density map obtained by in situ structural determination.

References

1. B. Martins *et al.,* Unveiling the polarity of actin filaments by cryo-electron tomography. *Structure* 10.1016/j.str.2020.12.014 (2021).
